# Safety Monitoring in Clinical Trials

**DOI:** 10.3390/pharmaceutics5010094

**Published:** 2013-01-17

**Authors:** Bin Yao, Li Zhu, Qi Jiang, H. Amy Xia

**Affiliations:** Amgen Inc., One Amgen Center Drive, Thousand Oaks, CA 91320, USA; E-Mails: zhul@amgen.com (L.Z.); qjiang@amgen.com (Q.J.); hxia@amgen.com (H.A.X.)

**Keywords:** clinical trial, safety monitoring, Data and Safety Monitoring Board (DSMB), sequential probability ratio test (SPRT), Bayesian methods

## Abstract

Monitoring patient safety during clinical trials is a critical component throughout the drug development life-cycle. Pharmaceutical sponsors must work proactively and collaboratively with all stakeholders to ensure a systematic approach to safety monitoring. The regulatory landscape has evolved with increased requirements for risk management plans, risk evaluation and minimization strategies. As the industry transitions from passive to active safety surveillance activities, there will be greater demand for more comprehensive and innovative approaches that apply quantitative methods to accumulating data from all sources, ranging from the discovery and preclinical through clinical and post-approval stages. Statistical methods, especially those based on the Bayesian framework, are important tools to help provide objectivity and rigor to the safety monitoring process.

## 1. Introduction

Clinical trials provide the evidentiary basis for regulatory approvals of safe and effective medicines. With long development cycles and ever-increasing costs in conducting clinical trials, both the pharmaceutical industry and regulators are making efforts to be more proactive in safety evaluations. Early safety signal detection not only leads to better patient protection, but also has the potential to save development costs. 

Since clinical trials are experiments in humans, they must be conducted following established standards in order to protect the rights, safety and well-being of the participants. These standards include the International Conference on Harmonization Good Clinical Practice (ICH-GCP) Guidelines [1], International Ethical Guidelines for Biomedical Research Involving Human Subjects issued by the Council for International Organizations of Medical Sciences (CIOMS) [2] and the ethical principles set forth in the Declaration of Helsinki [3]. GCP is the “standard for the design, conduct, performance, monitoring, auditing, recording, analyses and reporting of clinical trials that provides assurance that the data and reported results are credible and accurate and that rights, integrity and confidentiality of trial subjects are protected” [1]. The globalization of clinical trials has presented additional challenges to sponsors. Sponsors are held accountable to comply with the relevant local legal and regulatory requirements wherever the clinical trials are conducted. For example, clinical trials conducted in the European Union are required to be conducted in accordance with the Clinical Trials Directive [4]. 

Safety evaluation is a central component in all stages of the drug development lifecycle. Prior to the marketing authorization of a drug, rigorous safety monitoring and evaluations from preclinical to all stages of clinical trials are required. Pharmaceutical sponsors need to adequately characterize the safety profile of the product in order to obtain regulatory approval and marketing authorization. The approved product label contains the essential information about the product’s benefits and risks. The continued vigilance in safety is critical as more data and experience is gathered from a broader patient population once the product is on the market. In some cases, new emerging safety profiles may cast the original benefit-risk assessments in doubt. These are evidenced in some high profile market withdrawals, such as Troglitazone (Rezulin), Rofecoxib (Vioxx) and Rosiglitazone (Avandia). In 2005, the United States Food and Drug Administration (FDA) issued guidance documents on risk management activities, including premarket risk assessment and postmarketing pharmacovigilance and pharmacoepidemiologic assessments [5,6,7]. Regulatory agencies around the world and the pharmaceutical industry are taking a more comprehensive and holistic approach to safety evaluation in drug development. 

This article will focus on safety monitoring during the pre-approval period. Section 2 summarizes the common practice in safety monitoring in clinical trials. Relevant regulatory guidance and industry guidelines are discussed. In Section 3, quantitative safety monitoring methods based on statistical principles are presented. An example is provided to illustrate the applications. We conclude with discussion and recommendations.

## 2. Common Practice in Safety Monitoring

### 2.1. Stakeholders in Safety Monitoring

#### 2.1.1. Sponsor

Clinical trial sponsors, usually pharmaceutical companies, are responsible for developing the clinical trial protocol. The protocol describes every aspect of the research, including the rationale for the experiment, objectives, trial population with detailed inclusion and exclusion criteria, administration of the investigational therapies, trial procedures, data collection standards, endpoints and sample size. The protocol also details the safety reporting procedures, specifically on the requirements for expedited reporting of serious adverse events. The Informed Consent Form (ICF) is used to disclose current information about the investigational drug and about the procedures, risks and benefits for subjects who participate in the clinical trial. Informed consent is a vital part of the research process. In addition to the protocol and the ICF, sponsors are responsible for setting up and maintaining clinical databases for the data collected in the trial. Case Report Forms (CRFs) are designed by the sponsor as data collection tools. These tools are increasingly based on electronic data capture modules via the internet rather than the traditional paper-based route. With access to all accumulating data, sponsors are mandated to report key safety information to all stakeholders in a timely fashion. Details of the sponsor’s reporting requirements are discussed in Section 2.2. 

#### 2.1.2. Subjects

Subjects are patients or healthy volunteers who agree to participate in a clinical trial and have signed the ICF. Along with other information, the ICF provides important safety information so the subjects can make an informed decision on whether to participate in the trial. The informed consent must be given freely, without coercion and must be based on a clear understanding of what participation involves. By giving consent, subjects permit the investigators to collect health information and body measurements as per the protocol. While subjects are encouraged to follow the protocol to trial completion, they can withdraw at any time. They do not need to give a reason for withdrawing consent. In a phase 1 clinical trial, when the drug is first used in humans, healthy volunteers are compensated for their time and willingness to be exposed to unknown risks. Later phase trials are mostly conducted in patients with the disease of interest, and payments to these subjects for participation are contentious. The main concern is the payment could be coercive or serve as undue inducement leading to impaired judgment on trial participation [8]. 

#### 2.1.3. Investigators

Investigators are qualified individuals who are trained and experienced to provide medical care to subjects enrolled in the trial. Investigators identify potential subjects and educate them about the trial participation to ensure that they can make an informed decision. While the trial is ongoing, investigators are expected to adhere to the protocol treatment plan in delivering care. They observe, evaluate, manage and document all effects of treatment, including the reporting of adverse events. They are responsible for notifying their institutional review boards and the sponsor of any issues that pose a threat to the safety and well-being of the trial subjects. Investigators are ultimately accountable and responsible for the conduct of the clinical trial and for the safety of the subjects under their care. 

#### 2.1.4. Institutional Review Board/Ethics Committee

The Institutional Review Board (IRB), also known as the ethics committee, is charged with protecting the rights and welfare of human subjects recruited to participate in research protocols conducted under the auspices of the institution to which the IRB is affiliated. The IRB reviews all clinical trial protocols involving human subjects that the particular institution is involved with and has the authority to approve, disapprove or require modifications to the protocols. IRBs bear further the responsibility of reviewing ongoing research to ensure continued diligence that subjects are not placed at undue risk and they give uncoerced, informed consent to their participation. The training and education of investigators at the institution who participate in clinical research is also a responsibility of the IRB. Members of an IRB generally come from a wide range of scientific disciplines and from outside academic communities in which research is being conducted.

#### 2.1.5. Data and Safety Monitoring Board

The Data and Safety Monitoring Board (DSMB), also called data monitoring committee (DMC), is an expert committee, independent of the sponsor, chartered for one or more clinical trials. The mandate of the DSMB is to review on a regular basis the accumulating data from the clinical trial to ensure the continuing safety of current participants and those yet to be enrolled. The DSMB may review efficacy data at pre-defined interim points to assess whether there’s overwhelming evidence of efficacy or the lack thereof, such that the clinical equipoise at the beginning of the trial is no longer justified. DSMB has the additional responsibilities to advise the sponsor regarding the continuing validity and scientific merit of the trial. Not all clinical trials require a formal DSMB. DSMBs are most common in double blind randomized phase 3 trials. Members of the DSMB typically include clinical trial experts, including physicians with the appropriate specialty, at least one biostatistician and possibly person(s) from other disciplines, such as biomedical ethics, basic science/pharmacology or law. 

#### 2.1.6. Regulatory Authorities

In the US, prior to the initiation of a first in human clinical trial, pharmaceutical sponsors must submit an Investigational New Drug (IND) application to the FDA as required by law. The FDA reviews the IND (typically within 30 calendar days) for safety to ensure that research subjects will not be subjected to unreasonable risk. In 2010, the FDA issued guidance to sponsors and investigators on safety reporting requirements for human drug and biological products that are being investigated under an IND and for drugs that are the subjects of bioavailability (BA) and bioequivalence (BE) studies that are exempt from the IND requirements [9]. The guidance provided the agency’s expectations for timely review, evaluation and submission of relevant and useful safety information and implemented internationally harmonized definitions and reporting standards. The European Medicines Agency (EMA) is the European Union’s FDA equivalent. The agency has several scientific committees that carry out the evaluation of applications from pharmaceutical companies. In other parts of the world, regulatory authorities will have similar mandates, but may operate under different local laws and regulations. 

#### 2.1.7. Medical Community and Patients

Clinical trials generate data that contribute to the body of knowledge about the treatment and the disease that benefit the broader medical community and, ultimately, the patients. Safety information of one product may be informative to other practitioners using a similar class of agents. In 1997, the US Congress passed the Food and Drug Modernization Act (FDAMA), requiring clinical trial registration. ClinicalTrials.gov was created as a result. The website was further expanded in 2007 after the Congress passed the Food and Drug Administration Amendments Act (FDAAA), which required more types of trials to be registered. In September 2008, as required by FDAAA 801, ClinicalTrials.gov began allowing sponsors and principal investigators to submit the results of clinical studies. Submission of adverse event information was optional when the results database was released and became required in September 2009. The mandatory requirement on clinical trial registration and the disclosure of trial results are significant achievements in advancing science and increasing transparency in clinical research. 

### 2.2. Communicating Safety Information among Stakeholders

Timely communication among the various stakeholders is critical to ensure subject safety in clinical trials. Sponsors of clinical trials are accountable for monitoring the subjects appropriately, including the requirement of long-term follow up as appropriate. The protocol (including the ICF) specifies the details of the assessments, the frequency and the length of follow-up. In addition, most pharmaceutical sponsors have Standard Operating Procedures (SOPs) in place to collect, process, review, evaluate, report and communicate accumulating safety data to ensure a systematic approach for safety surveillance and monitoring. In general, safety information, including adverse events and laboratory findings are reported to a sponsor by investigators conducting the clinical trial. However, safety information may come from sources outside the immediate clinical trial. The sponsor is required to promptly review all information relevant to the safety of the drug and to update subjects, investigators, IRBs and regulatory authorities of any new risks associated with the use of the investigational drug that arise from the clinical trial or from other sources. 

Amending the clinical trial protocol is one way to implement procedural changes that are necessary given the updated safety information. Another way to communicate the evolving safety information is through the periodic update of the Investigator’s Brochure (IB). The IB is a compilation of the clinical and non-clinical data on the investigational drug that are relevant to the study of the drug in human subjects. Its purpose is to provide the investigators and others involved in the trial with the information to facilitate their understanding of the rationale for and their compliance with many key features of the protocol, such as the dose, dose frequency/interval, methods of administration and safety monitoring procedures [1]. The IB should be reviewed at least annually and revised as necessary in compliance with the sponsor’s written procedures and the local requirements. A new safety finding that represents a significant risk to study subjects should be communicated to the investigators immediately, along with an update to the IB and possibly to the protocol and the ICF. For trials where DSMBs are in place, sponsors should also communicate significant safety findings to the DSMBs employed to oversee clinical trials of the same or similar investigational drug(s). In other situations, DSMBs may be in possession of critical safety information and they will need to follow the DSMB charter and the protocol to make recommendations to the sponsor with regard to its safety findings and whether the trial should continue as planned. 

The goal of safety monitoring in clinical trials is to identify, evaluate, minimize and appropriately manage risks. In Europe, Risk Management Plans (RMPs) are required by the EMA as part of the drug approval process. An RMP includes a summary of important identified risks of the drug, potential risks and missing information, which serves as the basis for an action plan for pharmacovigilance and risk minimization activities. The CIOMS VI working group [10] recommended establishing a multidisciplinary Safety Management Team (SMT) within the sponsor organization to be in charge of safety surveillance and decision making on risk management and minimization activities. The SMT is responsible for coordinating all safety-related activities involving quantitative assessment of risks, signal detection and identification of adverse events of special interest (AESIs). For trials in earlier stages without DSMBs, sponsors may choose to appoint an internal multi-functional data review team removed from the direct day-to-day trial operations related to the investigational drug to perform ongoing review of the safety data. This independent data review team is empowered to perform similar functions as the DSMB on later stage trials. 

The CIOMS VI working group endorsed the use of the Development Core Safety Information (DCSI) as the summary of the identified safety issues for an investigational drug. DCSI was recommended to be a part of the IB that defines the list of suspected adverse reactions. Safety issues or adverse drug reactions contained in this document should be considered “expected” for regulatory reporting purposes. Only suspected adverse drug reactions that are both serious and unexpected are subject to expedited case reporting to regulatory authorities in either seven (fatal or life-threatening) or 15 calendar days. Slightly different terminologies exist, including Suspected Unexpected Serious Drug Reaction (SUSAR) [4] or Serious Unexpected Suspected Drug Reaction [9]. Contrary to the routine expedited case reporting to regulatory authorities, the CIOMS VI working group recommended sponsors provide periodic updates of the evolving benefit/risk profile and highlight important new safety information to the participating investigators and IRBs. However, in some regions, expedited case reporting to investigators and IRBs are still required by local regulations. 

Regulatory authorities also require reporting of safety information in the aggregate rather than the individual cases. In the US, the FDA IND regulations require annual IND reports, which include aggregate safety information across the entire development program of an investigational drug. CIOMS VI working group recommended defining a single Development Safety Update Report (DSUR) for submission to regulators on an annual basis. For submission of New Drug Applications (NDAs), sponsors aggregate safety information from all relevant trials of the drug to perform integrated safety analyses in support of the filing for marketing authorization. The common data structure using SDTM (Standard Data Tabulation Model) defined by Clinical Data Interchange Standards Consortium (CDISC) has greatly facilitated the safety data integration and analyses. It also enables sponsors to build a safety data warehouse to better respond to safety related queries across the entire drug program. Proactive early planning of safety analyses in a Program Safety Analysis Plan (PSAP) and periodic aggregate safety analyses have been recommended as standard industry practices [11,12]. The PSAP is a living document that will form the basis for integrated safety analyses in an NDA. 

## 3. Statistical Methods in Safety Monitoring

### 3.1. Methods for Single Arm Trials

With the continued focus on safety monitoring and increased amount of work on case processing and reporting, quantitative approaches to safety evaluations will become increasingly important. Statistical methods can be applied to set up objective criteria in safety assessments and to help detect signals hidden in the volume of safety data. Most phase 2 and phase 3 clinical trials are designed with treatment efficacy as the primary objectives. It is important to consider inclusion of safety assessment criteria in addition to the evaluation of efficacy. In practice, sponsors usually have some ideas about the safety profile based on the mechanism of action of the drug or based on data from preclinical/animal testing, previous trials or data from drugs in a similar class. We recommend sponsors establish upfront safety monitoring criteria to help guide the DSMBs (or the sponsor’s independent data review team) on serious adverse events, such as SUSARs or AESIs, even when the trial is not comparative. 

As an example, in oncology, it is not uncommon to have a single arm phase 2 trial where all subjects are treated with the same experimental agent. Consider a sequential analysis that the occurrence of an undesirable AESI is evaluated after every subject is treated for a fixed period of time. We assume the probability of the AESI in subjects treated with the agent is *π.* The sequential probability ratio test (SPRT) introduced by Wald [13,14] can be used to set up a monitoring scheme of the AESI. Let the null and alternative hypotheses be:

*H*_0_: π = π_0_ and *H*_1_: π = π_1_, (1)

where π_0_ represents the background event rate (*i.e.*, event rate expected of a standard control therapy) considered acceptable and π_1_ represent the event rate considered not acceptable.

Let *Y_i_* = *y_i_*, *i* = 1, 2, ... , *n*, be the binary indicator of whether the AESI is observed from the *i*-th subject. ***y_n_*** = (*y*_1_, *y*_2_, ... , *y_n_*)*^T^* denotes the vector of *n* subjects who have already been treated. The sequence of probability ratios (*i.e.*, likelihood ratios) is defined as:

Λ*_n_* = *f*(***y_n_***; π = π_1_)/*f*(***y_n_***; π = π_0_) (2)

Let *n*_max_ denote the total number of subjects planned for the trial. The monitoring guideline using SPRT is as follows:

- if Λ*_n_* ≥ *U_n_*, conclude π_1_;
- if Λ*_n_* ≤ *L_n_*, conclude π_0_;
- if *L_n_* < Λ*_n_* < *U_n_* and *n* < *n*_max_, then continue the trial.

The upper and lower boundaries in the above monitoring guideline can be solved by specifying the type I (false positive) and type II (false negative) error rates related to the hypothesis testing. The type I error here amounts to falsely identifying a safety problem when none exists, and the type II error means failing to identify a safety problem when one does exist. The type I error may be set at 0.05 and the type II error at 0.2.

While improvements and extensions to the SPRT have been proposed [15], we will examine instead an alternative approach based on the Bayesian framework by Thall and Simon (TS method) [16]. Assuming the prior distributions of *π* and *π*_S_ follow Beta distributions with π ~ Beta(a, *b*) and π_S_ ~ Beta(*a*_S_, *b*_S_), the parameters of the prior distributions can be determined based on either historical data or expert opinions [17]. Unlike in the SPRT, π_S_ is assumed to follow a distribution based on historical information of the standard control therapy. The posterior distribution of π upon observing *x_i_* events in the first *i* subjects (*i* = 1, ... , *n*_max_) is also a Beta distribution:

(π| *X_i_* = *x_i_*) ~ Beta(*a* + *x_i_*, *b* + *i* − *x_i_*) (3)

It should be noted that during the trial, the parameters characterizing the distribution of π get updated upon observing new data, while those of π_S_ remain the same, as there is no standard control therapy arm. For any *i* = 1, ... , *n*_max_, a criterion function is defined as follows:

ϕ(*x_i_*, *i*; π, π_S_, δ) = *P*(π – π_S_ > δ| *i*, *X_i_* = *x_i_*) (4)

where δ is a positive constant that is pre-specified. Let *U*_i_ be the smallest integer, such that ϕ(*U_i_* , *i*; π, π_S_, 0) ≥ *p_U_*, and *L*_i_(*<U_i_*) be the largest integer, such that ϕ(*L_i_*, *i*; π, π_S_, δ) ≤ *p_L_*, where *p_U_* with a large value (e.g., ≥0.9) and *p_L_* with a small value (e.g., ≤0.05) are predetermined threshold probabilities.

The monitoring guideline after the *i*-th subject has been treated is as follows:

- if *x_i_* ≥ *U_i_*, conclude *π g*reater than π_S_;
- if *x_i_* ≤ *L_i_*, conclude π less than π_S_ + δ;
- if *L_i_* < *x_i_* < *U_i_* and *i* < *n*_max_, then continue the trial.

The upper boundary implies that any treatment that is highly likely to incur an increased risk over standard treatment is of concern and may not warrant further development. The lower boundary implies that a treatment that is very unlikely to cause an increased risk of at least δ over π_S_ is considered tolerable.

It should be noted that the monitoring guidelines discussed above for the SPRT and the TS methods are for the general cases. When monitoring safety events, the lower boundaries are usually not relevant and can be ignored. Trials will be stopped when it is considered unsafe to continue. However, whether to stop a trial could depend on many factors, such as other safety observations and efficacy in the context of risk-benefit trade-off. In practice, monitoring guidelines may be considered after a minimum number of subjects have been treated to ensure reliable estimates can be obtained [18]. One important parameter in setting up a monitoring guideline is the specification of π_1_ in the SPRT method and δ in the TS method. We recommend close collaboration with clinical experts in soliciting realistic input. Computer simulation of potential outcomes given various scenarios can also help make a decision. 

### 3.2. Methods for Randomized, Controlled Trials

For the randomized, controlled clinical trials, we consider similar explicit statistical boundaries for safety monitoring. Here, the monitoring is based on the unblinded aggregate data reviewed regularly by either the DSMB or the sponsor’s independent data review team depending on the nature of the trial. We assume the monitoring involves an AESI as in Section 3.1. The event rate in the treated and control arms are π_T_ and π_C_, respectively.

We adopt a Bayesian Beta-Binomial model in setting up the monitoring. At any time point *t* during the trial, the prior distributions are assumed to be beta distributions with π*_T_* ~ Beta(*a_T_*, *b_T_* ) and π*_C_* ~ Beta(*a_C_*, *b_C_*). The posterior distributions can be expressed as:

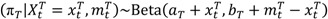
(5)

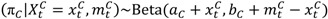
(6)
where 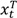 and 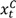 are the numbers of events from the treatment and the control arm and 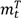 and 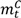 are the numbers of subjects on the treatment arm and the control arm up to time *t*. 

The criterion function is defined as follows:

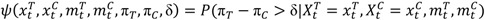
(7)
where δ is the pre-specified tolerable risk difference between the two arms on the AESI. When 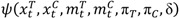 *> p*, where *p* is the predetermined upper threshold probability, for example, *p* = 0.9, the risk is considered alarming to warrant considerations of stopping the trial due to imbalance of toxicities. 

In situations when additional information regarding exposure in the form of person-time is available, Bayesian methods with the Gamma-Poisson model may be applied instead of the Beta-Binomial model.

### 3.3. A Hypothetical Clinical Trial Example

We apply the methods discussed in Section 3.1 and Section 3.2 to a hypothetical clinical trial. Suppose there is a randomized, double blind, controlled clinical trial with planned sample size *n*_max_ = 120. Subjects will be randomized in a 1:1 ratio to the investigational drug arm or the control arm. Based on previous knowledge about the disease and the biological mechanism of action of the investigational drug, the team feels that a serious AESI is a potential risk and requires heightened surveillance. The trial has an independent DSMB. 

We first consider a situation where the sponsor monitors the event of the AESI from the combined treatment arms. Suppose an increasing number of observed AESI is reported amidst rapid enrolment, but the next scheduled DSMB meeting is months away. By closely monitoring the overall event rate in the combined arms, the sponsor may be able to decide whether an ad hoc DSMB meeting is warranted. If the pre-determined monitoring boundary is crossed, the team may call the DSMB into an ad hoc session to avoid any delays in waiting for the next scheduled DSMB meeting. Assume the rate of the event from the combined group is deemed acceptable if it’s no more than 5% and unacceptable if it is greater than 21%. The boundary to alert the DSMB using the SPRT method is derived using 0.05 type I error and 80% power. The boundary values for the first 10 events are shown in Table 1. For example, if three events of the AESI are observed in 11 or fewer subjects, the boundary is crossed and the team may consider convening an ad hoc DSMB meeting. The boundary values for SPRT can be obtained from the online calculation tool [19].

**Figure 1:**
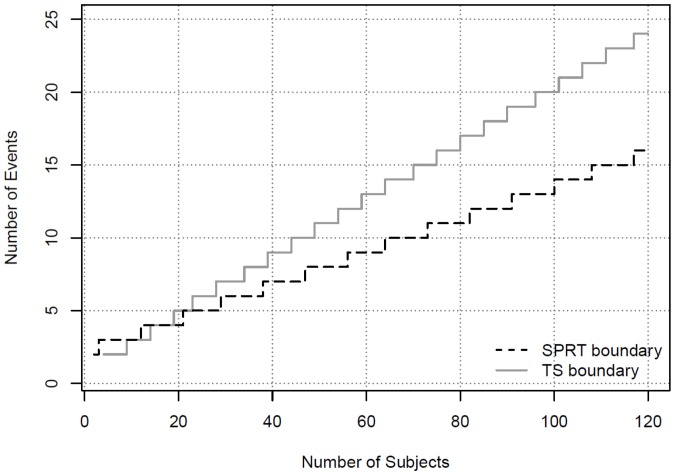
Safety Boundaries Using Blinded Data.

In addition to the SPRT, Bayesian methods can be used if some knowledge is available to form the prior distributions. We apply the TS method and assume the prior for the AESI in the control arm follows a Beta distribution with Beta(3, 57), and the prior for the AESI in the combined treatment and control group follows Beta(3, 11). Note that the distribution of Beta(3, 57) represents information from prior experiences equivalent to three events from 60 subjects. Similarly, Beta(3, 11) represents prior information equivalent to three events out of 14 subjects. Our choices for the prior distributions assume more knowledge about the control group from historical data, but less knowledge about the experimental group given limited experience. The criterion to alert the DSMB can be established as *P*(π – π_S_ > 0.1| *i, X_i_* = *x_i_*) > 0.9. The derivation of the Bayesian safety boundary follows Section 3.1 or by using the free software Multc Lean, downloadable from the MD Anderson site [20]. The boundary values based on the Bayesian method for the first 10 events are shown in Table 1. They are also plotted in Figure 1. It can be seen that the SPRT method has boundary values that are easier to cross in declaring a safety concern than the Bayesian method, except when only two events are observed. 

**Table 1:** Safety Boundary Using Blinded Data.

	Number of Subjects	
**Number of Events**	**SPRT**	**TS**
1	n.a.	n.a.
2	2	≤3
3	≤11	≤8
4	≤20	≤13
5	≤28	≤18
6	≤37	≤22
7	≤46	≤27
8	≤55	≤33
9	≤63	≤38
10	≤72	≤43

We now consider setting up the monitoring boundary for the DSMB who has access to the actual treatment assignments. For the same AESI, the Bayesian Beta-Binomial method in Section 3.2 can be used. Assuming the prior distributions are π_T_ ~ Beta(3, 11) and π_C_ ~ Beta(3, 57) and applying the stopping criterion 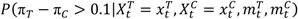 > 0.9, the operating characteristics for such a monitoring guideline can be computed through simulations. 

The Bayesian boundaries when monitoring the two arms cannot be easily plotted, but the DSMB can use a posterior probability table as a reference. In this example, there are eight subjects in the treatment arm and 11 subjects in the control arm at the analysis time. Table 2 shows the posterior probability table that summarizes the values of the criterion function 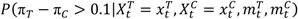 under all possible scenarios. In this table, all the bolded numbers highlight the scenarios in which the stopping boundary is crossed. For instance, if at this analysis time there are 3 subjects on the treatment arm who experience the AESI, while none of the subjects on the control arm has the AESI, then 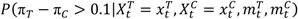 = 0.92 and the boundary is crossed.

**Table 2:** An Example of Posterior Probability Table.

		**Treatment Arm: Events/Subjects**							
		1/8	2/8	3/8	4/8	5/8	6/8	7/8	8/8
**Control Arm: Events/Subjects**	0/11	0.65	0.82	**0.92**	**0.97**	**0.99**	**1**	**1**	**1**
	1/11	0.58	0.77	0.89	**0.96**	**0.98**	**1**	**1**	**1**
	2/11	0.52	0.71	0.85	**0.94**	**0.97**	**0.99**	**1**	**1**
	3/11	0.45	0.65	0.81	**0.91**	**0.96**	**0.99**	**1**	**1**
	4/11	0.39	0.59	0.76	0.88	**0.94**	**0.98**	**0.99**	**1**
	5/11	0.34	0.53	0.71	0.84	**0.93**	**0.97**	**0.99**	**1**
	6/11	0.29	0.47	0.66	0.81	0.90	**0.96**	**0.98**	**0.99**
	7/11	0.24	0.42	0.60	0.76	0.87	**0.94**	**0.97**	**0.99**
	8/11	0.21	0.37	0.55	0.71	0.84	**0.92**	**0.96**	**0.99**
	9/11	0.17	0.32	0.50	0.67	0.80	0.90	**0.95**	**0.98**
	10/11	0.14	0.27	0.44	0.61	0.76	0.87	**0.93**	**0.97**
	11/11	0.11	0.23	0.39	0.56	0.72	0.84	**0.92**	**0.96**

## 4. Conclusions

Monitoring patient safety during clinical trials is a critical component throughout the drug development life-cycle. Pharmaceutical sponsors must work proactively and collaboratively with all stakeholders to ensure a systematic approach to safety monitoring. The regulatory landscape has evolved with increased requirements for risk management plans, risk evaluation and minimization strategies. As the industry transitions from passive to active safety surveillance activities, there will be greater demand for more comprehensive and innovative approaches that apply quantitative methods to accumulating data from all sources, ranging from the discovery and preclinical through clinical and post-approval stages.

We have discussed several statistical methods that can be applied to monitor ongoing clinical trials in either a blinded or an unblinded fashion. We recommend the Bayesian approach as the analytical framework for safety monitoring due to its flexibilities in incorporating the ‘current’ knowledge of the safety profile into the decision making. In addition, Bayesian methods allow sponsors to take advantage of information originating from multiple sources both internal and external to the trial. This is an important advantage, as safety signals identified in clinical trials alone may be limited. The globalization of clinical trials has posed additional challenges. A great deal of coordination is required of sponsors to ensure timely communication of new safety findings among all stakeholders in all regions. Efforts in building a standard safety data warehouse across all trials in a development program will lay a solid foundation for integrated safety analyses. Innovative statistical methods can be applied to increase the efficiency in reviewing a large volume of safety data, to identify safety trends and to establish prospective monitoring guidelines, as described in this article. 
